# A pilot study to determine the timing and effect of bevacizumab on vascular normalization of metastatic brain tumors in breast cancer

**DOI:** 10.1186/s12885-016-2494-8

**Published:** 2016-07-13

**Authors:** Bang-Bin Chen, Yen-Shen Lu, Ching-Hung Lin, Wei-Wu Chen, Pei-Fang Wu, Chao-Yu Hsu, Chih-Wei Yu, Shwu-Yuan Wei, Ann-Lii Cheng, Tiffany Ting-Fang Shih

**Affiliations:** Department of Medical Imaging and Radiology, National Taiwan University College of Medicine and Hospital, Taipei City, Taiwan; Department of Oncology, National Taiwan University College of Medicine and Hospital, Taipei City, Taiwan; Department of Radiology, Taipei Hospital, Ministry of Health and Welfare, New Taipei City, Taiwan; Department of Medical Imaging, Taipei City Hospital, Taipei City, Taiwan

**Keywords:** Bevacizumab, DCE-MRI, Breast cancer, Chemotherapy

## Abstract

**Background:**

To determine the appropriate time of concomitant chemotherapy administration after antiangiogenic treatment, we investigated the timing and effect of bevacizumab administration on vascular normalization of metastatic brain tumors in breast cancer patients.

**Methods:**

Eight patients who participated in a phase II trial for breast cancer-induced refractory brain metastases were enrolled and subjected to 4 dynamic contrast-enhanced magnetic resonance imaging (DCE-MRI) examinations that evaluated *Peak*, *Slope*, *iAUC*_*60*_, and *Ktrans* before and after treatment. The treatment comprised bevacizumab on Day 1, etoposide on Days 2–4, and cisplatin on Day 2 in a 21-day cycle for a maximum of 6 cycles. DCE-MRI was performed before treatment and at 1 h, 24 h, and 21 days after bevacizumab administration.

**Results:**

Values of the 4 DCE-MRI parameters reduced after bevacizumab administration. Compared with baseline values, the mean reductions at 1 and 24 h were −12.8 and −24.7 % for *Peak*, −46.6 and −65.8 % for *Slope*, −27.9 and −55.5 % for *iAUC*_*60*_, and −46.6 and −63.9 % for *Ktrans*, respectively (all *P* < .05). The differences in the 1 and 24 h mean reductions were significant (all *P* < .05) for all the parameters. The generalized estimating equation linear regression analyses of the 4 DCE-MRI parameters revealed that vascular normalization peaked 24 h after bevacizumab administration.

**Conclusion:**

Bevacizumab induced vascular normalization of brain metastases in humans at 1 and 24 h after administration, and the effect was significantly higher at 24 h than at 1 h.

**Trial registration:**

ClinicalTrials.gov, identifier NCT01281696, registered prospectively on December 24, 2010

## Background

Angiogenesis has been a therapeutic target in treating several solid tumor types for decades [1–4]. Tumor vascular normalization has recently been proposed as an alternative to antiangiogenesis [5]. Tumor vasculature is generally dysfunctional and comprises tortuous, dilated, and leaky vessels that lead to elevated interstitial pressure and adversely affect drug delivery [6]. Animal studies have shown that antiangiogenic therapy can normalize the abnormal structures and functions of tumor blood vessels and improve drug delivery [7–9]. In clinical settings, a combination of bevacizumab, an antivascular endothelial growth factor (VEGF) monoclonal antibody, and chemotherapy has been used for treating metastatic breast cancer [10]. However, the modest effect of bevacizumab observed in subsequent studies resulted in the withdrawal of its indications by the Food and Drug Administration [11].

Thus, we considered the vascular normalization theory and hypothesized that bevacizumab preconditioning and subsequent chemotherapy is more effective than the current standard treatment, wherein bevacizumab and chemotherapy are concomitantly used. This hypothesis was supported by an animal study in which chemotherapy was administered 1–3 days after bevacizumab administration; the chemotherapy penetration improved by approximately 81 %, resulting in increased tumor growth inhibition compared with that of concomitant chemotherapy and bevacizumab administration [12]. In addition, bevacizumab administration 1 day before etoposide and cisplatin administration appeared highly effective in patients with breast cancer whose brain metastases progressed after whole-brain radiotherapy (WBRT) [13]. The central nervous system (CNS) tumor objective response rate was 77.1 % according to the volumetric criteria, and the median CNS progression-free survival (PFS) and overall survival duration were 7.3 and 10.5 months, respectively [13]. Although the unexpected high efficacy strongly supports our hypothesis, understanding whether vascular normalization occurs immediately or 24 h after bevacizumab administration is crucial. This information can facilitate identifying the appropriate time for administering chemotherapeutic agents following bevacizumab treatment in humans. Dynamic contrast-enhanced magnetic resonance imaging (DCE-MRI) can facilitate noninvasive determination of the contrast agent leakage kinetics from the vasculature [14] and is a suitable technique for assessing bevacizumab treatment response [15]. Therefore, we used DCE-MRI for evaluating bevacizumab-induced vascular responses in patients with metastatic brain tumors originating from the breast.

## Methods

### Patient characteristics and clinical outcomes

This prospective study was approved by the institutional review board of National Taiwan University Hospital, and written informed consent was obtained from all study participants before enrollment. Between January 2011 and January 2013, we conducted a multicenter phase II study, in which patients with breast cancer whose brain metastases progressed after WBRT were enrolled. The patients were intravenously administered 15 mg/kg of bevacizumab for 90 min on day 1, etoposide at 70 mg/m^2^/day from day 2– to day 4, and 70 mg/m^2^ of cisplatin on Day 2 (hereafter, the BEEP regimen) in a 21-day cycle for a maximum of 6 cycles. The response assessment criteria, including tumor objective response rate and PFS, were described previously (registered at ClinicalTrials.gov, identifier NCT01281696) [13]. Eight patients agreed to participate in a serial DCE-MRI study, the optional translational research stage of the phase II study. The patients underwent 4 DCE-MRI examinations as follows: before bevacizumab treatment, 1 ± 0.5 h after the completion of bevacizumab administration (2.5 h after starting bevacizumab application, which is the time at which chemotherapeutic agents are conventionally administered), 24 ± 2 h after starting bevacizumab administration, and 21 days after the BEEP regimen was administered.

### Magnetic resonance imaging protocol

The participants fasted for 4 h and rested in the supine position in the MR scanner. MRI was performed using a 3.0-T superconducting magnet (Magnetom Verio; Siemens Medical Systems, Erlangen, Germany) with an 8-channel head coil and applying the axial precontrast T1-weighted turbo spin echo sequence (TR/TE, 4/1.2 ms; flip angle, 150°; matrix, 232 × 256; field of view, 181 × 200 mm; and slice thickness/interslice gap, 4/1.2 mm).

Subsequent quantification was performed using a 3D gradient-echo sequence with isotropic resolution in all three brain dimensions. A T1 brain map was initially created using six flip angles (2, 5, 10, 15, 20, and 25°) to determine the baseline precontrast values for the dynamic procedure. An MR pulse sequence of T1-weighted volumetric interpolated brain examination (TR/TE, 6/2.5 ms; flip angle, 18°; matrix, 232 × 256; field of view, 208 × 230 mm; slice thickness/interslice gap, 3/0 mm; temporal resolution per volume, 5.49 s; and z-axis coverage, 104 mm with center on the target lesion) was initiated 50 s before the injection of a standard dose (0.1 mmoL/kg body weight) of gadobutrol (Gd-BT-DO3A, Gadovist®, Bayer Schering, Berlin, Germany) at a flow of 3 mL/s, followed by a 50-mL saline flush at the same flow rate. Eighty volumes were acquired in a total measurement time of 6 min and 23 s. Postcontrast axial T1-weighted image sequences, which were identical to the precontrast image sequences, were obtained after DCE-MRI.

### Tumor volumetric measurement

Tumor volumetric measurement was performed by an experienced radiologist who was blinded to the treatment status of the patients. All the enhanced lesions on post-contrast T1-weighted images were outlined using a volumetric approach, which outlined each enhancing voxel on postcontrast scans and then summed the voxels to calculate an overall lesion volume [16].

### Data postprocessing

Postprocessing of all DCE-MRI data was performed using a commercial software tool (MIStar; Apollo Medical Imaging, Melbourne, Australia) for image segmentation and coregistration [17]. The slice with the largest diameter in a target lesion was selected and measured in operator-defined regions of interest by an experienced radiologist to obtain a time-signal intensity curve.

Three semiquantitative parameters (*Peak*, *Slope*, and *iAUC*_*60*_) were determined [17, 18]. *Peak* was defined as (SI_max_ − SI_base_)/SI_base_ × 100, where SI_base_ was the average baseline signal before the inflow of the contrast agent in the arteries and SI_max_ was the maximal value of the first pass of the time-signal intensity curve. *Slope* was derived from the steepest part of the first-pass portion of the time-signal intensity curve. *iAUC*_*60*_ (mM · s) was the initial area under the time-signal intensity curve within 60 s of contrast inflow. *Ktrans* (1/min) was derived using a bicompartmental model of Tofts et al. [19] and through nonlinear fitting of individual time-signal intensity curves. These parameters were automatically calculated pixel by pixel from the fitted curve. To measure the arterial input function, a region of interest was defined in the middle cerebral artery. *Ktrans* was related to the permeability surface product per unit volume of extravascular extracellular space in nonflow-limited situations.

### Statistical analysis

Data are expressed as means and standard deviations (SDs). In univariate analysis, the relative changes in DCE-MRI parameters at 1 h, 24 h, and 21 days were determined by comparing the final parameter values with baseline values. Subsequently, multivariate analysis was performed by fitting multiple linear regression models to identify predictors of the relative changes in the 4 DCE-MRI parameters over time. Generalized estimating equations (GEEs) [20] were used for determining the correlations between repeated measurements of each patient. The statistical power of the data was analyzed using repeated-measurement ANOVA. A 2-sided *P* ≤ .05 was considered statistically significant. Statistical analysis was performed using SPSS 15.0 (SPSS Inc., Chicago, Illinois, USA) and R 3.0.2 (R Foundation for Statistical Computing, Vienna, Austria).

## Results

### Patient characteristics, tumor response, and clinical outcomes

The median age of the participants was 49.2 years (range: 35.7–71.8 y). Three patients were estrogen receptor (ER) positive and human epidermal growth factor receptor 2 (HER2) negative, one patient was ER and HER2 positive, and four patients were ER negative and HER2 positive (Table 1). The median number of extra-CNS metastasis sites and BEEP protocol treatment cycles was 2.5 (range: 1–3) and six (range: 3–6), respectively. CNS lesions of all patients exhibited partial responses at 9 weeks, and five patients subsequently underwent MRI to confirm the CNS objective response. With a median follow-up of 16.8 months, the median CNS-specific PFS time was 9.1 months (95 % confidence interval [CI], 4.7–13.5), and the overall survival time was 10.7 months (95 % CI, 7.8–18.8). The mean CNS tumor size at the baseline was 29.5 ± 37.4 cm^3^. The average change in the lesion size from baseline was −43.6 ± 18.5 % at 21 days, −56.2 ± 12.1 % at 9 weeks (*N* = 8), and −66.7 ± 16.7 % at 18 weeks (*N* = 6).

**Table 1:** Demography and clinical characteristics of patients (*n* = 8)

Characteristics	Number	Percent
Age		
Median (range)	49.2 (35.7–71.8)	
Histology Type		
Invasive Ductal Carcinoma	8	100
Hormone Receptor Status		
ER- and PR-	3	37.5
ER- and PR+	4	50.0
ER+ and PR-	0	0
ER+ and PR+	1	12.5
HER2 Expression		
IHC 0-2+ and/or FISH -	3	37.5
IHC 2+ and FISH +, IHC 3+	5	62.5
Hormonal and HER2 Status		
Triple Negative	0	0.0
ER+ and HER2-	3	37.5
HER2+	5	62.5
Molecular Subtype		
Luminal A	3	37.5
Luminal B	1	12.5
HER2+/ER-	4	50.0
Basal-like	0	0.0
ECOG Performance Status		
0-1	4	50.0
2	3	37.5
3	1	12.5

Note: *ER* estrogen receptor, *PR* progesterone receptor, *HER2* human epidermal growth factor receptor 2, *IHC* immunohistochemistry, *FISH* fluorescent in situ hybridization, *ECOG* Eastern Cooperative Oncology Group

### Change in dynamic contrast-enhanced magnetic resonance imaging parameters in the first cycle of BEEP

**Fig. 1:**
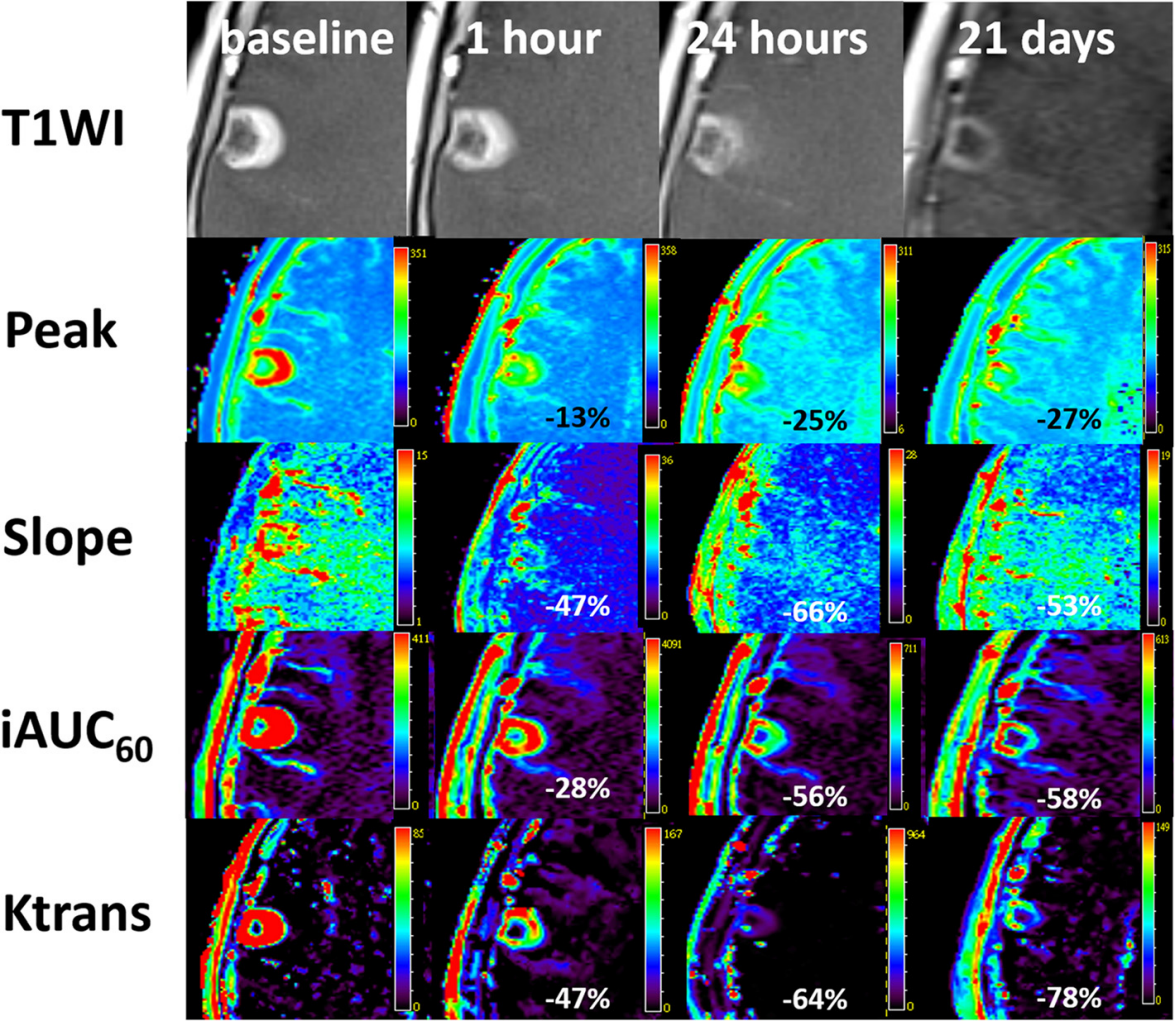
A 75-year-old patient with breast cancer with a single brain metastasis had a partial response after 3 BEEP regimen cycles. T1-weighted image (T1WI) and DCE-MRI parameter maps (*Peak*, *Slope*, *iAUC*_*60*,_ and *Ktrans*) of the metastatic tumor in the right frontal lobe at baseline, 1 h, 24 h, and 21 days after the first cycle of BEEP regimen, respectively

**Fig. 2:**
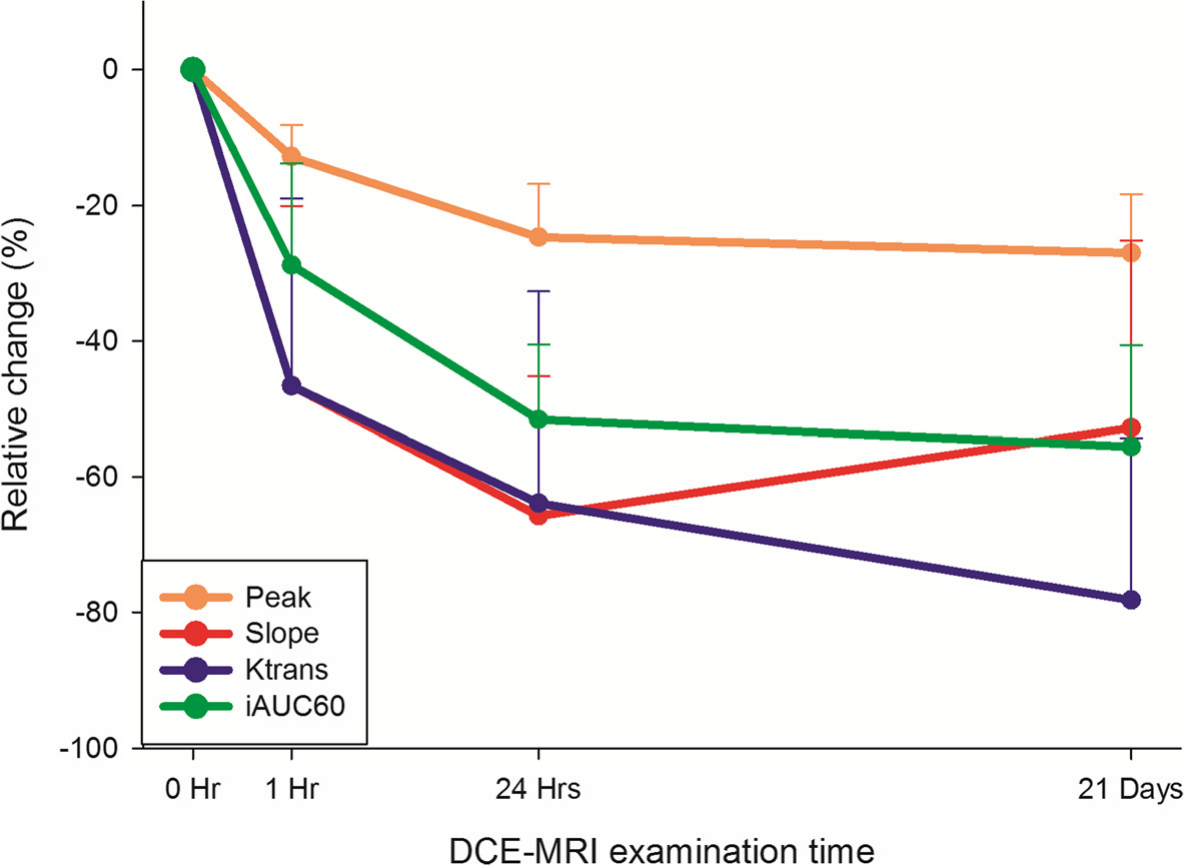
Mean percentage changes of DCE-MRI parameters at 1 h, 24 h, and 21 days compared with baseline values

Compared with the baseline values, the reductions in the mean percentage change of all 4 DCE-MRI parameters at 1 h, 24 h, and 21 days were significantly different from zero (Table 2, Figs. 1 and 2), which indicated decreased gross angiogenesis within tumors after the BEEP treatment. Moreover, the reductions in the mean percentage change of all 4 DCE-MRI parameters were significantly higher at 24 h than at 1 h, which suggested higher effect of vascular normalization of bevacizumab at 24 h than at 1 h. The differences between *Peak*, *Slope*, *iAUC*_*60*_, and *Ktrans* values at 1 and 24 h were −13.4 ± 10.1 % (*P* = .023), −31.7 ± 24.0 % (*P* = .008), −30.0 ± 20.0 % (*P* = .008), and −40.6 ± 30.6 % (*P* = .023), respectively, as per the Wilcoxon signed-rank test. However, the differences in the mean percentage change between 24 h and 21 days were nonsignificant for all 4 parameters (*P* > .05). The GEE linear regression analyses for the 4 DCE-MRI parameters indicated that the extent of vascular normalization was maximal at 24 h among three time points (1 h, 24 h and 21 days) after bevacizumab administration.

**Table 2:** Relative changes in DCE-MRI parameters compared with baseline values at 1 h, 24 h, and 21 days after the first dose of bevacizumab in eight patients with breast cancer with brain metastases

Parameters	Baseline	Δ1 h(%)	Δ24 h(%)	Δ21 Days(%)
*Peak*	223.3 ± 47.2	−12.8 ± 4.6	−24.7 ± 7.9	−27 ± 8.6
*Slope*	1.5 ± 1.1	−46.6 ± 26.5	−65.8 ± 20.6	−52.8 ± 27.7
*iAUC*_*60*_	49820 ± 31258	−27.9 ± 15	−55.5 ± 11.1	−58.1 ± 15
*Ktrans (min* ^*-1*^ * x 1000)*	858.1 ± 1194.7	−46.6 ± 27.6	−63.9 ± 31.2	−78.2 ± 23.9

Data are presented as the mean ± standard deviations (SD). The Wilcoxon signed-rank test had *P* < .01 compared with the baseline DCE-MRI data

The repeated-measurement ANOVA analysis for the percentage changes of these repeated measurements (percentage changes between pretreatment and 1 h, between pretreatment and 24 h, and between pretreatment and 21 days) were significantly different (*P* < .01 for Greenhouse-Geisser and Huynh-Feldt tests) with high statistical power (> .9) for *Peak, iAUC*_*60*,_ and *Ktrans* but not for *slope* (*P* > .05). The results suggested that DCE-MRI parameters were helpful to evaluate serial changes of tumor angiogenesis after anti-angiogenic agents with high statistical power.

## Discussion

We used DCE-MRI for evaluating bevacizumab-induced tumor vascular responses in humans. All DCE-MRI parameters exhibited significant reductions as early as 1 h after the completion of bevacizumab administration, which is the time at which chemotherapeutic agents are conventionally administered. However, maximal reduction was observed at 24 h. Thus, bevacizumab-induced vascular normalization is time dependent, and bevacizumab administration at least 24 h before chemotherapy may substantially enhance cytotoxic activity by increasing drug delivery to the tumor tissue.

The potential mechanisms underlying DCE-MRI parameter changes are reduction in tumor permeability, perfusion, or volume through effective administration of cytotoxic agents. Because only bevacizumab was administered on the first day, the reduction from the baseline to 24 h can solely be attributable to the bevacizumab effect. Studies have indicated that vascular normalization is a major outcome of bevacizumab use, and the breast tumor is unlikely to shrink after sole bevacizumab treatment [12, 21]. Therefore, the change in DCE-MRI parameters from the baseline to 24 h most likely resulted from the normalization of tumor vasculature rather than tumor mass change. By contrast, the parameter changes on Day 21 may have resulted from a combination of the antiangiogenesis effect of bevacizumab and the antitumor effect of cytotoxic agents. These complex effects may explain why the differences in the mean percentage changes of the 4 parameters between 24 h and 21 days were nonsignificant.

We used three semiquantitative model-free parameters (*Peak*, *Slope*, and *iAUC*_*60*_) to evaluate brain tumor perfusion [22]. *Peak* was the concentration of the contrast agent in the intravascular and extravascular extracellular spaces and indicated the sum of the vessel density and permeability factors [23]. *Slope* indicated the concentration of the contrast agent in the intravascular space and can be determined according to tissue vascularization, perfusion, and capillary permeability [19, 24]. *iAUC*_*60*_ was correlated with blood flow, vessel permeability, and interstitial space [25]. These model-free metrics are not based on specific physiology and most likely represent a combination of tumor blood flow, blood volume, and permeability [18]. By contrast, the model-based quantitative parameter, *Ktrans*, reflects the rapid transport of the contrast agent from the plasma to the extravascular space and is a function of both permeability and vessel surface area [26]. The consistent decrease in all 4 DCE-MRI parameters from the baseline to 24 h further validated our vascular normalization hypothesis. In addition, based on our data, Ktrans was the best parameter for clinical use among 4 DCE-MRI parameters because its relative change was larger than other three parameters after antiangiogenic treatment, and thus may be more sensitive to detect vascular normalization and treatment response.

*Ktrans* values facilitate evaluating the response to antiangiogenic agents, predicting tumor recurrence or progression, and determining the optimal time at which the blood–brain barrier opens to the maximum extent during treatment [27–29]. *Ktrans* is dependent on both the permeability of capillaries and blood flow in the tumor tissue. When the capillary permeability is high, *Ktrans* is equal to the blood plasma flow per unit volume of tissue, whereas when the blood flow is high, *Ktrans* is equal to the product of the permeability and the surface area of the capillary vascular endothelium [30]. Thus, *Ktrans* may be highly dependent on blood flow because of the high permeability of the disorganized vasculature in untreated tumors. However, this parameter becomes permeability dependent after bevacizumab treatment because one consequence of vascular normalization is increased blood flow.

In our previous report, bevacizumab administration 1 day before etoposide and cisplatin administration was highly effective in treating patients with breast cancer whose brain metastases progressed after WBRT [13]. Although our study did not enroll patients who received concurrent chemotherapy with bevacizumab for comparison, we compared our results with those of Lin et al. [31], who used a conventional dosing schedule, in which carboplatin was administered immediately after bevacizumab administration in breast cancer patients with brain metastasis. The baseline condition of patients, administration of whether other concomitant anticancer drugs, and treatment duration differed between these two studies. Lin et al. recruited only patients with an Eastern Cooperative Oncology Group (ECOG) performance status of ≤2, and 87 % patients were ECOG 0–1. In our study, 31 % of the patients had an ECOG of 3, and 23 % were ECOG 2, suggesting the difference in the baseline tumor burden between these 2 studies. In Lin et al., 22.6 % patients did not receive prior WBRT, whereas 100 % of our patients received prior WBRT and their brain tumors progressed after WBRT. In Lin et al., protocol treatment was continued until disease progression, whereas in our study, patients only received a maximum of 6 cycles (4 months) of protocol treatment because of budget limitation. All HER2-positive patients received trastuzumab in addition to bevacizumab and carboplatin in the study of Lin et al., whereas none of the HER2-positive patients in our study received trastuzumab during the 6 cycles of the BEEP regimen. The overall results revealed that compared with concurrent use, our study achieved a higher tumor response and longer PFS with sequential use in patients with breast cancer, even though our patient group seemed to have more advanced disease status than that of Lin et al. Additional confirmatory studies are necessary for direct comparison of the efficacy between bevacizumab pretreatment 1 day before chemotherapy and immediate sequential use of bevacizumab and chemotherapy in patients with cancer.

To investigate the enhancement of drug delivery to the CNS by bevacizumab-induced vascular normalization, we measured the etoposide concentration in the cerebrospinal fluid (CSF); however, we found that bevacizumab exerts no significant effects on CSF drug concentrations [32]. Microdialysis is used for measuring drug concentrations in the brain parenchyma; however, this procedure cannot be readily used in clinical trials because of ethical reasons [32]. The assumptions that CSF readily equilibrates with brain interstitial fluid and CSF drug concentrations reflect drug concentrations in the brain parenchyma have remained debatable. The choroid plexus, which regulates drug transfer into the CSF from the blood, and the brain capillary endothelium, which regulates drug transfer into the interstitial fluid from the blood, comprise completely different epithelial or endothelial barriers. Studies have demonstrated different transporter expression profiles in the blood–CSF and the blood–brain barriers, supporting the opinion that the drug concentration in the CSF can significantly deviate from that in the brain parenchyma [33, 34].

Positron emission tomography (PET) with 18-fluorodeoxyglucose (^18^F-FDG) can evaluate tumor metabolism and is applied for diagnosis, staging and monitoring of cancer. ^18^F-FDG has been evaluated as imaging biomarker that can predict and assess responses to various types of therapies including conventional chemotherapeutic drugs and newer targeted anti-cancer therapies in various tumor types [35]. For example, by dynamic ^18^F-FDG PET analyses, treatment with bevacizumab was shown to reduce both the tumor perfusion and metabolism 24 h post-treatment in triple-negative breast cancer xenografts [36]. FDG uptake was also prognostic of response to bevacizumab-based therapy in recurrent high-grade glioma [37]. Recently, hybrid PET/MR systems provide advantages of combined imaging of brain tumor metabolism and perfusion, and may offer complementary information on tumor biology and monitor changes after treatment [38].

This study had several limitations. First, the sample size was small. Although there were only eight patients, concerns regarding lack of power usually arise when known or plausible results are not confirmed or not detected in statistical testing, which was not the case in this study. All eight patients exhibited consistent and significant changes after the first cycle of bevacizumab administration. Second, we measured the DCE-MRI parameters only in the largest target tumor. We assumed that the perfusion changes were maximum in larger tumors than in smaller tumors after chemotherapy, and we compared the same tumor longitudinally because considering multiple tumors involves high variability. Third, no pathological specimen was available for direct histological examination of the change in the vasculature and interstitial pressure after bevacizumab treatment at different time events in our study. A recent study reported that the addition of bevacizumab to chemotherapy in patients with triple-negative breast cancer significantly increases pathological complete response rates [39]. A study that correlates DCE-MRI parameters with pathological response rates after bevacizumab administration may facilitate delineating the relationship between vascular normalization and treatment effect. Forth, Willett et al. found a significant increase in tumor cell apoptosis at day 12 after bevacizumab administration [40, 41] in rectal cancer patients, but we did not perform DCE-MRI at day 12 due to concern of MR contrast agent dose by our institutional review board. In addition, several additional studies may help explore the underlying mechanisms of antivascular effect of bevacizumab [42–44]. For examples, we may use plasm soluble vascular endothelial growth factor-1 [44] to evaluate the change of tumor oxygenation before and after bevacizumab treatment and correlate this potential biomarker with DCE-MRI parameters.

## Conclusion

In conclusion, after anti-VEGF therapy with bevacizumab, vascular normalization can occur as early as 1 h after bevacizumab administration, and the effect is more prominent after 24 h. Our study strongly indicated that pretreatment with bevacizumab for a long duration can enhance the efficacy of chemotherapy compared with the conventional concurrent use of bevacizumab and chemotherapy. Additional studies are warranted to determine the optimal time for administering antiangiogenesis therapy and chemotherapy in clinical practice.

## Abbreviations

CNS, central nervous system; CR, complete response; DCE-MRI, dynamic contrast–enhanced magnetic resonance imaging; ER, estrogen receptor; GEE, generalized estimating equations; HER2, human epidermal growth factor receptor 2; PD, progressive disease; PFS, progression-free survival; PR, partial response; SD, standard deviations; VEGF, vascular endothelial growth factor

## References

[1] Senger DR, Galli SJ, Dvorak AM, Perruzzi CA, Harvey VS, Dvorak HF (1983). Tumor cells secrete a vascular permeability factor that promotes accumulation of ascites fluid. Science.

[2] Leung DW, Cachianes G, Kuang WJ, Goeddel DV, Ferrara N (1989). Vascular endothelial growth factor is a secreted angiogenic mitogen. Science.

[3] Folkman J (1971). Tumor angiogenesis: therapeutic implications. N Engl J Med.

[4] Kim KJ, Li B, Winer J, Gillett N, Phillips HS, Ferrara N (1993). Inhibition of vascular endothelial growth factor-induced angiogenesis suppresses tumour growth in vivo. Nature.

[5] Inai T, Mancuso M, Hashizume H, Baffert F, Haskell A, Baluk P (2004). Inhibition of vascular endothelial growth factor (VEGF) signaling in cancer causes loss of endothelial fenestrations, regression of tumor vessels, and appearance of basement membrane ghosts. Am J Pathol.

[6] Sitohy B, Nagy JA, Dvorak HF (2012). Anti-VEGF/VEGFR therapy for cancer: reassessing the target. Cancer Res.

[7] Jain RK (2001). Normalizing tumor vasculature with anti-angiogenic therapy: a new paradigm for combination therapy. Nat Med.

[8] Jain RK (2005). Normalization of tumor vasculature: an emerging concept in antiangiogenic therapy. Science.

[9] Ma J, Waxman DJ (2008). Combination of antiangiogenesis with chemotherapy for more effective cancer treatment. Mol Cancer Ther.

[10] Chan A, Miles DW, Pivot X (2010). Bevacizumab in combination with taxanes for the first-line treatment of metastatic breast cancer. Ann Oncol.

[11] Reddy S, Raffin M, Kaklamani V (2012). Targeting angiogenesis in metastatic breast cancer. Oncologist.

[12] Dickson PV, Hamner JB, Sims TL, Fraga CH, Ng CY, Rajasekeran S (2007). Bevacizumab-induced transient remodeling of the vasculature in neuroblastoma xenografts results in improved delivery and efficacy of systemically administered chemotherapy. Clin Cancer Res.

[13] Lu YS, Chen TW, Lin CH, Yeh DC, Tseng LM, Wu PF (2015). Bevacizumab preconditioning followed by etoposide and cisplatin is highly effective in treating brain metastases of breast cancer progressing from whole-brain radiotherapy. Clin Cancer Res.

[14] Hylton N (2006). Dynamic contrast-enhanced magnetic resonance imaging as an imaging biomarker. J Clin Oncol.

[15] O’Connor JP, Jackson A, Parker GJ, Roberts C, Jayson GC (2012). Dynamic contrast-enhanced MRI in clinical trials of antivascular therapies. Nat Rev Clin Oncol.

[16] Sorensen AG, Patel S, Harmath C, Bridges S, Synnott J, Sievers A (2001). Comparison of diameter and perimeter methods for tumor volume calculation. J Clin Oncol.

[17] Chen BB, Hsu CY, Yu CW, Wei SY, Kao JH, Lee HS (2012). Dynamic contrast-enhanced magnetic resonance imaging with Gd-EOB-DTPA for the evaluation of liver fibrosis in chronic hepatitis patients. Eur Radiol.

[18] Chung WJ, Kim HS, Kim N, Choi CG, Kim SJ (2013). Recurrent glioblastoma: optimum area under the curve method derived from dynamic contrast-enhanced T1-weighted perfusion MR imaging. Radiology.

[19] Tofts PS, Brix G, Buckley DL, Evelhoch JL, Henderson E, Knopp MV (1999). Estimating kinetic parameters from dynamic contrast-enhanced T(1)-weighted MRI of a diffusable tracer: standardized quantities and symbols. J Magn Reson Imaging.

[20] Liang KYZS (1986). Longitudinal data analysis using generalized linear models. Biometrika.

[21] Lambrechts D, Lenz H-J, de Haas S, Carmeliet P, Scherer SJ (2013). Markers of response for the antiangiogenic agent bevacizumab. J Clin Oncol.

[22] Narang J, Jain R, Arbab AS, Mikkelsen T, Scarpace L, Rosenblum ML (2011). Differentiating treatment-induced necrosis from recurrent/progressive brain tumor using nonmodel-based semiquantitative indices derived from dynamic contrast-enhanced T1-weighted MR perfusion. Neuro Oncol.

[23] Chen BB, Hsu CY, Yu CW, Hou HA, Liu CY, Wei SY (2011). Dynamic contrast-enhanced MR imaging measurement of vertebral bone marrow perfusion may be indicator of outcome of acute myeloid leukemia patients in remission. Radiology.

[24] Verstraete KL, Van der Woude HJ, Hogendoorn PC, De-Deene Y, Kunnen M, Bloem JL (1996). Dynamic contrast-enhanced MR imaging of musculoskeletal tumors: basic principles and clinical applications. J Magn Reson Imaging.

[25] Evelhoch JL (1999). Key factors in the acquisition of contrast kinetic data for oncology. J Magn Reson Imaging.

[26] Leach MO, Brindle KM, Evelhoch JL, Griffiths JR, Horsman MR, Jackson A (2005). The assessment of antiangiogenic and antivascular therapies in early-stage clinical trials using magnetic resonance imaging: issues and recommendations. Br J Cancer.

[27] Jain R (2013). Measurements of tumor vascular leakiness using DCE in brain tumors: clinical applications. NMR Biomed.

[28] Batchelor TT, Sorensen AG, di Tomaso E, Zhang WT, Duda DG, Cohen KS (2007). AZD2171, a pan-VEGF receptor tyrosine kinase inhibitor, normalizes tumor vasculature and alleviates edema in glioblastoma patients. Cancer Cell.

[29] Sorensen AG, Batchelor TT, Zhang WT, Chen PJ, Yeo P, Wang M (2009). A “vascular normalization index” as potential mechanistic biomarker to predict survival after a single dose of cediranib in recurrent glioblastoma patients. Cancer Res.

[30] Miller JC, Pien HH, Sahani D, Sorensen AG, Thrall JH (2005). Imaging angiogenesis: applications and potential for drug development. J Natl Cancer Inst.

[31] Lin NU, Gelman RS, Younger WJ, Sohl J, Freedman RA, Sorensen AG, et al. Phase II trial of carboplatin (C) and bevacizumab (BEV) in patients (pts) with breast cancer brain metastases (BCBM). J Clin Oncol. 2013;31(suppl; abstr 513). http://meetinglibrary.asco.org/content/83798?media=vm.

[32] Wu PF, Lin CH, Kuo CH, Chen WW, Yeh DC, Liao HW (2015). A pilot study of bevacizumab combined with etoposide and cisplatin in breast cancer patients with leptomeningeal carcinomatosis. BMC Cancer.

[33] Kamiie J, Ohtsuki S, Iwase R, Ohmine K, Katsukura Y, Yanai K (2008). Quantitative atlas of membrane transporter proteins: development and application of a highly sensitive simultaneous LC/MS/MS method combined with novel in-silico peptide selection criteria. Pharm Res.

[34] Kusuhara H, Sugiyama Y (2004). Efflux transport systems for organic anions and cations at the blood-CSF barrier. Adv Drug Deliv Rev.

[35] Jensen MM, Kjaer A (2015). Monitoring of anti-cancer treatment with (18)F-FDG and (18)F-FLT PET: a comprehensive review of pre-clinical studies. Am J Nucl Med Mol Imaging.

[36] Kristian A, Revheim ME, Qu H, Mælandsmo GM, Engebråten O, Seierstad T (2013). Dynamic (18)F-FDG-PET for monitoring treatment effect following anti-angiogenic therapy in triple-negative breast cancer xenografts. Acta Oncol.

[37] Colavolpe C, Chinot O, Metellus P, Mancini J, Barrie M, Bequet-Boucard C (2012). FDG-PET predicts survival in recurrent high-grade gliomas treated with bevacizumab and irinotecan. Neuro Oncol.

[38] Henriksen OM, Larsen VA, Muhic A, Hansen AE, Larsson HB, Poulsen HS (2016). Simultaneous evaluation of brain tumour metabolism, structure and blood volume using [(18)F]-fluoroethyltyrosine (FET) PET/MRI: feasibility, agreement and initial experience. Eur J Nucl Med Mol Imaging.

[39] Gerber B, Loibl S, Eidtmann H, Rezai M, Fasching PA, Tesch H (2013). Neoadjuvant bevacizumab and anthracycline-taxane-based chemotherapy in 678 triple-negative primary breast cancers; results from the geparquinto study (GBG 44). Ann Oncol.

[40] Willett CG, Boucher Y, Duda DG, di Tomaso E, Munn LL, Tong RT (2005). Surrogate markers for antiangiogenic therapy and dose-limiting toxicities for bevacizumab with radiation and chemotherapy: continued experience of a phase I trial in rectal cancer patients. J Clin Oncol.

[41] Willett CG, Duda DG, di Tomaso E, Boucher Y, Ancukiewicz M, Sahani DV (2009). Efficacy, safety, and biomarkers of neoadjuvant bevacizumab, radiation therapy, and fluorouracil in rectal cancer: a multidisciplinary phase II study. J Clin Oncol.

[42] Willett CG, Boucher Y, di Tomaso E, Duda DG, Munn LL, Tong RT (2004). Direct evidence that the VEGF-specific antibody bevacizumab has antivascular effects in human rectal cancer. Nat Med.

[43] Gross S, Gilead A, Scherz A, Neeman M, Salomon Y (2003). Monitoring photodynamic therapy of solid tumors online by BOLD-contrast MRI. Nat Med.

[44] Duda DG, Willett CG, Ancukiewicz M, di Tomaso E, Shah M, Czito BG (2010). Plasma soluble VEGFR-1 is a potential dual biomarker of response and toxicity for bevacizumab with chemoradiation in locally advanced rectal cancer. Oncologist.

